# Underwater Logging: Submarine Rediscovers Lost Wood

**DOI:** 10.1289/ehp.112-a892

**Published:** 2004-11

**Authors:** David J. Tenenbaum

The global market for industrial wood products (including wood and paper) is a $400 billion industry, according to *From Forests to Floorboards: Trends in Industrial Roundwood Production and Consumption*, a 2001 report from the World Resources Institute. The report notes that real prices for timber rose 30% between 1975 and 1996, an increase that author Emily Matthews says indicates that demand is growing faster than supply. Worldwide demand for wood products is expected to grow steadily, according to the Food and Agriculture Organization of the United Nations. As the world’s thirst for wood grows and the resulting deforestation contributes to a wide range of environmental problems, one enterprising group has gone to a surprising location to search for more sustainable wood supplies—under the water.

A great amount of timber sank during log drives or was flooded during the construction of hydroelectric dams around the world. Although under water, the trees may be as good as new. One obvious—but dangerous and expensive—way to retrieve this “rediscovered wood” is to hire divers to run underwater saws. A second solution, uprooting the trees with a chain, mucks up the water and disrupts aquatic ecosystems.

Now Triton Logging, a firm in British Columbia, has come up with a third alternative: the Sawfish. This remotely piloted submarine—named for a relative of the shark that has a beak like a giant hedge trimmer—sports a long, electric-powered chain saw. Triton president Chris Godsall, who has a master’s degree in business and sustainability, had worked salvaging individual sunken logs when he realized there was more to gain by salvaging whole drowned forests. The Sawfish, he says, represents “an arranged marriage of marine and logging technologies” that may offer a sustainable way to reduce the environmental impacts of logging and the attendant road building.

## Underwater Logging: Tough, but not Impossible

Underwater logging is possible because many submerged trees and logs are barely affected by their decades of submersion. Lake and river water is often too cold and too deficient in oxygen for decay organisms to survive. (Ironically, the above-water portion of trees often must be discarded due to degradation by sunlight and microorganisms.)

Studies of logs raised from Lake Superior show slight color changes, but “the properties are virtually the same as modern timber,” says Terry Mace, who has studied underwater log retrieval for the Wisconsin Department of Natural Resources. And although sugars have leached from the Lake Superior logs, this effectively seasons the wood, making it highly desirable for use in musical instruments.

It’s hard to pinpoint how many trees are available for underwater logging. Some underwater logs were sunk or otherwise lost during log drives on rivers, but the majority came from forests submerged during the building of dams. The number of large dams—those more than 15 meters high—has increased nearly sevenfold since 1950, reported the World Resources Institute in *World Resources 2000–2001*. And while dam building has decreased sharply in developed countries due to environmental considerations and a lack of good sites, it does continue elsewhere. Godsall estimates that about 35,000 square kilometers of forest worldwide have already been submerged by dams. In British Columbia alone, he says, about 20 million trees lay underwater.

Although all that submerged timber seems like a waste, Godsall says the schedule and economics of dam building are to blame—the trees are considered expendable, and the costs of removing them are too high. Further, he says, “if you were to clear two hundred square miles of forest, where would it go? Could you cut it economically? Cost–benefit analyses done time and time again, [in Canada], in the States, in Russia, in Brazil, or Southeast Asia focus on [generation of] electricity, not logs, and the result is flooded forests.”

## One Sharp Fish

The Sawfish is 6 feet high, nearly 12 feet long, and 6 feet wide; it weighs 7,700 pounds. The craft is tethered to a cable carrying electric power, video feeds, and control circuits. A sonar system and eight onboard video cameras allow the sub to “fly very easily through the lake, without touching the lake floor,” says Godsall.

Staying off the bottom reduces the amount of silt that gets suspended in the water, Godsall says. He adds, “We don’t think we can sell a wood product that has some environmental benefits for terrestrial forests while fouling the aquatic environment. And we don’t like turbidity, which interferes with our visibility.”

The operator works in a control booth on a barge, directing the robot to the base of a standing tree. A hydraulically powered grapple (driven by vegetable oil, not hydraulic fluid) grabs the tree, and the sub screws a large air bladder to the trunk and inflates it. After the Sawfish saws the trunk with its 40-horsepower electric chain saw, the bladder lifts the tree to the surface. Workers then remove the bladder and the tree’s limbs. In three hours, the Sawfish can cut 37 trees.

The logs are then sent to a conventional lumber mill for processing. Although the drowned trees contain more moisture than living trees, the lumber can be air- or kiln-dried with little trouble, according to Triton.

Triton is progressing toward certification under the Rediscovered Wood underwater salvage standards established by SmartWood, a nonprofit environmental program of the Rainforest Alliance that began assessing the environmental, social, and economic impact of forestry operations in 1989. SmartWood assessors evaluate the negative and positive effects of an operation on the environment: what types of fluids and chemicals are used in the machine (in case there is a hose break), whether the operation creates a disturbance at the lake bottom, whether sediment is being disturbed, whether there is shoreline erosion where the logs are being removed, and whether the waterway is improving or worsening because of the operation.

The assessor then scores each criterion within the principal standards. A weak performance will result in conditions that must be met by the company before certification is granted. SmartWood also requests monitoring by the company of the environmental impact that the salvage operation has, and will ask to see the results of the monitoring during annual on-site audits, which are a requirement of certification.

## Cost versus Benefits

In logging, as in all natural resource industries, the cost of raw materials is critical, and the success of underwater wood will probably depend on economics, says Eugene Wengert, a forest products industry consultant and retired professor of forest ecology and management from the University of Wisconsin–Madison. The question, he says, is whether those trees are cheaper to cut than fresh ones. “Logging and sawmilling are not done because we really love it,” he says. “They are done to make a profit.”

Companies such as Timeless Timber of Ashland, Wisconsin, recover logs that sank during log drives on rivers as much as a century ago. The process of raising these river logs is expensive, so the company charges a premium for its wood, limiting its market to customers who appreciate the wood’s historic and environmental value.

But Godsall maintains that the Sawfish is not necessarily more expensive than normal cutting. “There should not be a premium on owning a thing you believe in,” he says. “Ninety-nine percent of everything that comes out of [a submerged] forest goes into established markets [at normal market prices].” At the same time, flooded forests can also contain some premium wood, he adds.

Triton is entering the regular market for so-called saw logs (logs large enough to mill into lumber), Godsall says. The company’s present output, mainly strong, desirable Douglas fir, is sold to mills making lumber for flooring, furniture, and construction. In August 2003 the first Sawfish began cutting trees in Lois Lake, British Columbia, a dam impoundment built in the 1930s to power a sawmill. A second Sawfish is under construction.

Given the enormous amount of flooded forestland, Triton plans to both use the Sawfish itself and sell the remote-controlled loggers to other companies at a cost of US$1 million and up. “There are millions and millions of trees underwater in our own backyard, and we’re addressing those with our logging operations,” says Godsall. “But there are underwater forests all around the world that are out of our reach.”

## The Environmental Payoff

Drowned logs, sunken trees, and wood from building demolition are all considered rediscovered wood. The environmental promise of using rediscovered wood is to reduce the impact of logging and the attendant road building on forests.

Roads allow an influx of invasive species, and they increase erosion and runoff to surface waters. And roads are very common in forests. According to a 2000 report from the National Center for Policy Analysis titled *Banning Roads, Burning Forests*, the National Forest System has over 383,000 miles of roads—eight times the mileage of the interstate highway system—on its 192 million acres. Most of these roads were built for timber harvesting, but have since been adopted by recreational forest visitors.

But the environmental benefits of recovering drowned trees are difficult to compare to the standard of being “sustainable” because the trees are not replaced, even though other trees are being drowned by newer dams. Underwater logging can pose an environmental hazard if silt on the drowned logs is distributed into the water. Unlike sawing, the past practice of yanking standing trees from lake beds can pollute the water with sediment, blocking the light needed by aquatic plants.

Some in the forest products industry question the need for underwater logging, noting that forests have been expanding for about a century in the United States. Wengert argues that conventional logging itself may actually be sustainable. “No way are we running out of wood,” he says. “We may be running out of some species, but since 1909, the supply of wood has been increasing [in the United States]. Most forests are sustainable, if you look widely enough. If you look at one county, maybe they will cut half the county. But if you look at a big enough area, [those trees are replaced elsewhere].” Unless it’s developed, he says, forestland continues as forestland.

Perhaps, but Godsall argues that sustainable forestry is still a goal, not a reality, in the industry. “The industry has changed tremendously in the last ten years,” he says. “We have seen a huge capacity building in forestry companies regarding environmental sustainability and responsible engagement [in social issues related to forestry]. But that capacity to understand the issues is not always converted into a collective approach to sustainable forestry.”

SmartWood’s William Timpano, who monitors the movement of certified wood through the production process so manufacturers can place SmartWood’s Rediscovered Wood logo on their products, points out another benefit. “Since this volume of timber is not natural,” he says, “taking the timber out may make for better fish habitat or increase the presence of naturally occurring aquatic fauna.”

With underwater logging, every acre of drowned trees that is chain sawed in a hydroelectric reservoir should translate into an acre of forest that’s left standing. And that, in turn, could translate into significant environmental benefits for the world.

## Figures and Tables

**Figure:**
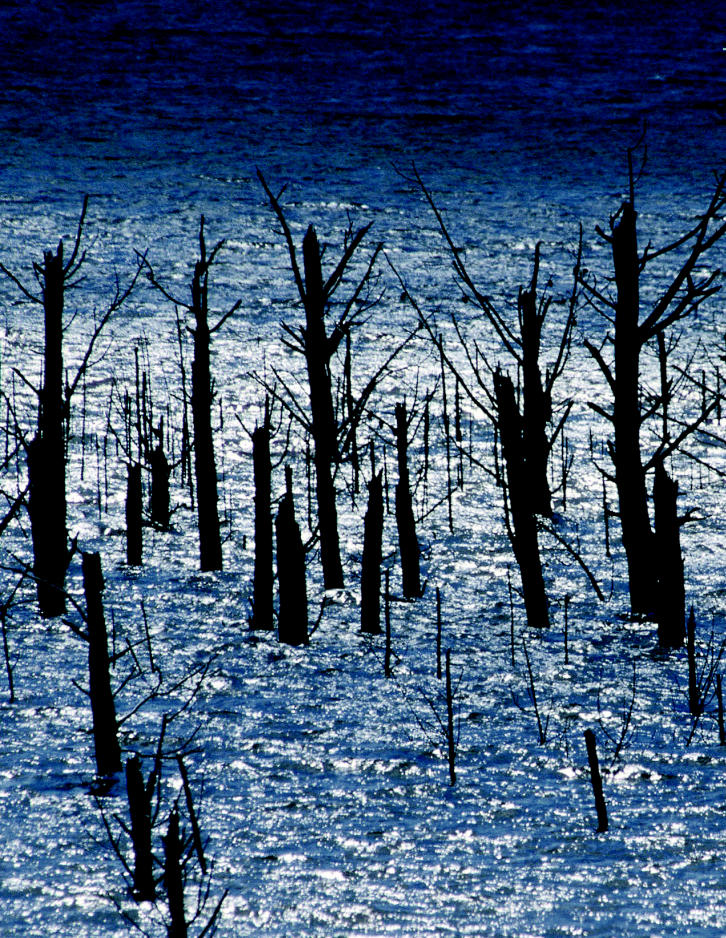


**Figure:**
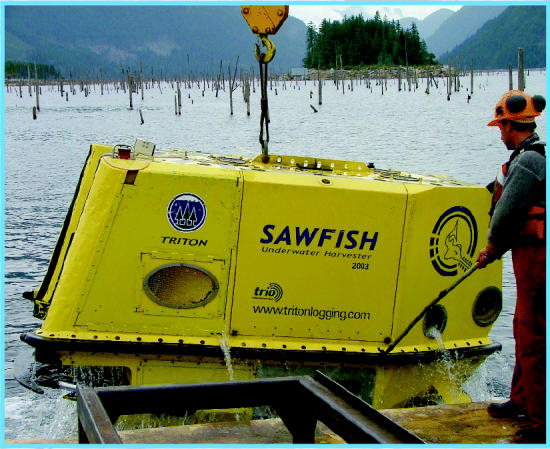


**Figure:**
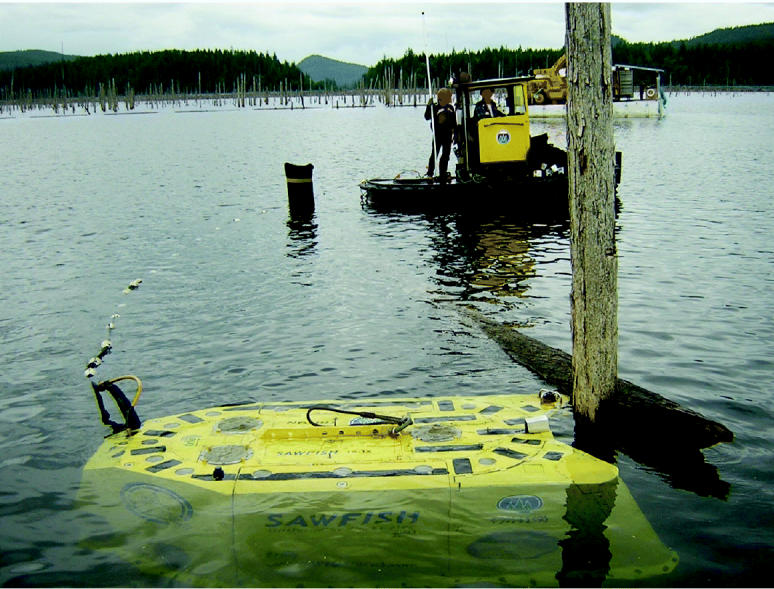

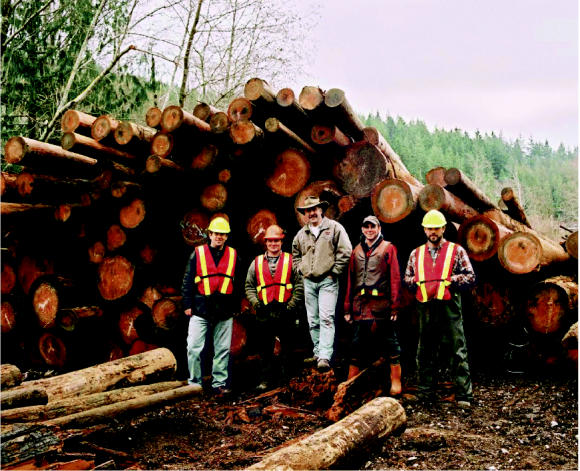
**Catch of the day.** The Sawfish in action (above), felling a submerged tree. The crew (left) stands in front of timber recovered from Lois Lake, British Columbia, which was flooded to create a reservoir.
